# Comparative evaluation of enzymatic, acidic, and alkaline digestion protocols for organic matrix removal from lyophilized and frozen mussel samples (*Mytilus galloprovincialis*)

**DOI:** 10.3389/ftox.2026.1808944

**Published:** 2026-04-15

**Authors:** S. Turmanova, Y. Hristov, D. Kiryakova, E. Ivanova, P. Atanasova, G. Kolchakova, A. Ilieva, E. Mollova, A. Dimitrov, N. Todorov, G. Grigorova

**Affiliations:** 1 Department of Materials Science, Burgas State University Prof. Dr. Assen Zlatarov, Burgas, Bulgaria; 2 Department of Ecology and Environmental Protection, Burgas State University Prof. Dr. Assen Zlatarov, Burgas, Bulgaria; 3 Department of Chemical Technologies, Burgas State University Prof. Dr. Assen Zlatarov, Burgas, Bulgaria; 4 Department of Biotechnology, Burgas State University Prof. Dr. Asen Zlatarov, Burgas, Bulgaria

**Keywords:** black sea, Burgas bay, digestion protocols, microplastics, *mytilus galloprovincialis*, recovery

## Abstract

Efficient removal of organic matter from biological matrices is a critical step in the analysis of microplastics in marine organisms. In this study, enzymatic (Kreon®25,000), acidic (HNO_3_ + H_2_O_2_), and alkaline (KOH + H_2_O_2_) digestion protocols were comparatively evaluated for the treatment of lyophilized and frozen *Mytilus galloprovincialis* samples. Digestion efficiency was assessed gravimetrically, while the effects of the protocols on polymer integrity were examined using ATR-FTIR, HQI analysis, and microscopic observations on representative polymers (HDPE, PA, PET, PVC). All three methods demonstrated high digestion efficiencies (>96%). Acidic digestion provided rapid and stable removal of organic matter within 20 min, whereas enzymatic digestion required longer incubation times (2–24 h) but exerted the least impact on polymer integrity. Frozen samples consistently showed slightly higher digestion efficiencies compared to lyophilized ones, likely due to preserved tissue hydration facilitating reagent penetration. Microscopic and spectroscopic analyses revealed that HDPE and PET maintained their structural and chemical integrity under all treatments, whereas PA and PVC exhibited surface alterations after acidic digestion. Enzymatic and alkaline protocols did not produce visible or spectral changes in any polymer type. Based on these findings, the enzymatic protocol was selected for recovery experiments. Mass-corrected recovery values ranged from 92.87% to 95.36% for PA, PET, and PVC, and 75.69% for HDPE, indicating that the method allows effective isolation of most polymers while preserving their integrity. The results demonstrate that although all digestion methods are efficient in removing organic matter, enzymatic digestion provides the most reliable approach for microplastic analysis in *Mytilus galloprovincialis*, ensuring both high digestion efficiency and preservation of polymer characteristics.

## 1 Introduction

Plastic waste entering the marine environment fragments under the influence of abiotic and biotic fragments into microplastics (MPs), defined as particles smaller than 5 mm (Cózar et al., 2014). Due to their persistence and ability to bioaccumulate, these particles can spread throughout marine ecosystems and enter food webs, becoming a potential threat to both marine biota and human health (Marcharla et al., 2024). Among marine organisms, filter-feeding bivalves, and particularly the black mussel *Mytilus galloprovincialis*, are especially vulnerable to MPs accumulation due to their feeding strategy, as they ingest both food particles and suspended contaminants that can be retained in the digestive system, gills, and soft tissues (Li et al., 2019; Dambrosio et al., 2023). In the present study, high-density polyethylene (HDPE), polyamide (PA), polyethylene terephthalate (PET), and polyvinyl chloride (PVC) were selected as representative polymers because they are among the most frequently detected microplastics in marine environments and in bivalve species, including *Mytilus galloprovincialis*. These polymers originate from common sources such as packaging materials (HDPE and PET), fishing gear (PA), and industrial or construction products (PVC).

In recent years, increasing attention has been paid to understanding the degradation of biological matter, especially with regard to the potential release of accumulated contaminants such as microplastics. Sample preparation through digestion of biotic material is a key step for the identification and quantitative analysis of MPs in marine organisms. As a widely used bioindicator for microplastic pollution (Li et al., 2019; Kovačić et al., 2024), *Mytilus galloprovincialis* requires complete or near-complete degradation of organic tissue to enable isolation and characterization of plastic particles without causing damage or chemical modification to them (Pfeiffer and Fischer, 2020).

Previous studies reported in the scientific literature indicate that the main laboratory approaches for removing biological matter during microplastic isolation are chemical in nature. Among these, enzymatic, acidic, alkaline, and oxidative treatments are commonly applied (Rani et al., 2023; Dehaut et al., 2016; Zhu and Wang, 2020; Rafiq and Xu, 2023; Schrank et al., 2022; Prata et al., 2019). Enzymatic protocols use lipolytic and proteolytic enzymes that degrade proteins and lipids in biological matter at moderate temperatures. They are characterized by minimal impact and preservation of the morphology and chemical composition of polymers (Cole et al., 2014; Catarino et al., 2017; Courtene-Jones et al., 2017). For example, an optimized digestion protocol using Proteinase K achieved over 97% removal of biological material from marine samples (Cole et al., 2014). Comparative studies of digestion procedures for mussel soft tissues demonstrated that complete digestion can be achieved using either 1 M NaOH, 35% HNO_3_, or the industrial protease Corolase®7089 at 60 °C for approximately 12 h (Catarino et al., 2017). However, HNO_3_ was found to degrade certain types of microplastics, while polymer recovery was similar for NaOH and enzymatic treatments - approximately 93% ± 10% (Catarino et al., 2017).

Other enzymes such as trypsin, papain, and collagenase exhibit varying efficiencies, with trypsin achieving up to 88% degradation within 30 min at 40 °C without damaging MPs during extraction from biological samples (Courtene-Jones et al., 2017). von Friesen et al. (2019) demonstrated that pancreatic enzymes (lipase, amylase, and protease) in Tris-HCl buffer (0.05 M, pH 8.0) can remove mussel soft tissues overnight with an efficiency of 97.7% ± 0.2% and a recovery rate of 87% ± 5.9%. In comparison, KOH treatment achieved only 33.1% ± 5.5% digestion and 75% ± 11.5% recovery, highlighting the strong dependence of alkaline protocol efficiency on sample condition, reagent concentration, and incubation conditions. For microplastic extraction from biota, a combined protocol involving rapid protein hydrolysis with pepsin (2 h) followed by alkaline hydrolysis (4 h), both at 37 °C, has also been applied (Süssmann et al., 2021). Only minor degradation of eleven commercial polymers was observed, with the exception of polyacrylonitrile (Süssmann et al., 2021). These findings confirm that enzymatic digestion is generally the preferred method, particularly when preservation of MPs structural properties is essential for subsequent analysis. However, its main limitations include variable efficiency (Dehaut et al., 2016), longer processing times (ranging from several hours to days), and the need for careful optimization of pH and temperature conditions (Löder et al., 2017).

Chemical digestion using acids and/or bases is also widely utilized in analytical protocols for the separation of microplastics from biological matter. These methods provide rapid and efficient degradation, with the recovery of polymer particles such as polyethylene (PE), polypropylene (PP), and polystyrene (PS) reaching 95%–100% (Tuttle and Stubbins, 2023). The process of acidic digestion of biota is typically performed using mineral acids, of which hydrochloric (HCl) and nitric (HNO_3_) acids are the most frequently applied in protocols reported in the literature (Pfeiffer and Fischer, 2020; Zhu and Wang, 2020; Schrank et al., 2022; Cole et al., 2014; Tuttle and Stubbins, 2023; Davidson and Dudas, 2016). Cole et al. (2014) found that treating marine samples with 1 M and 2 M HCl resulted in relatively low efficiency (82.6% ± 3.7% and 72.1% ± 9.2%), while alkaline treatment achieved values of 90.0% ± 2.9% and 85.0% ± 5.0% with 1 M and 2 M NaOH, respectively. The application of HNO_3_ (at room temperature overnight, followed by 2 h of boiling) ensures over 98% removal of organic matter from mussel biotic tissues (Claessens et al., 2013), primarily carbohydrates, proteins, and fats (Rani et al., 2023; Lusher et al., 2017). However, high acid concentrations and applied temperatures destroy Nylon fibers and cause the fusion of PS microbeads post-treatment (Claessens et al., 2013). Only 4% of PE and PS (4% ± 3%) were successfully recovered after treatment with 22.5 M HNO_3_. Complete elimination of PET and 95%–100% recovery of PE were observed after exposure to boiling 15.7 M HNO_3_ for 2 h (Schrank et al., 2022). Aung et al. (2022) confirm that HNO_3_ completely (100%) degrades organic tissue, but PE microplastic particles melt during this process. Simultaneously, enzymatic hydrolysis in combination with H_2_O_2_ and 5% HCl treatment showed low degradation and recovery efficiency. Satisfactory recovery levels of blue PE microplastics were achieved using H_2_O_2_ and KOH.

Tests involving various acidic and oxidative protocols were conducted by Desforges et al. (2015), including the use of: 100% HCl (12.1 M), a 1:1 v/v mixture of HCl and HNO_3_ (15.9 M), 100% HNO_3_, 100% H_2_O_2_ (0.9 M), and 1:1 v/v of HCl and H_2_O_2_ at room temperature and with heating (approximately 80 °C) for 1 h and 3 h. The results indicate that none of the mixtures completely eliminated zooplankton tissue at room temperature, regardless of the treatment duration. Treatment with nitric acid alone led to complete tissue removal upon heating, with no observed differences between the 3-h and 1-h treatments. The mixture of hydrochloric and nitric acids digested the zooplankton into small organic fragments that remained in the solution. Despite the high efficiency, the use of HNO_3_ and other strong acids in microplastics analysis is not recommended due to their corrosive effects and their capacity to cause partial or complete damage to certain polymers (Dehaut et al., 2016; Cole et al., 2014; Claessens et al., 2013; Aung et al., 2022). While the use of an oxidative solution Dehaut et al. (2016) has a minimal impact on plastics, its application results in the incomplete digestion of mussel tissues.

To accelerate the degradation process of organic matter, the effect of HNO_3_ is combined with oxidizing agents such as hydrogen peroxide, H_2_O_2_ (Bianchi et al., 2020; Yu et al., 2019). Bianchi et al. (2020) investigated the stability of microplastics in mixtures of HNO_3_ (in concentrations ranging from 5% to 55%) and H_2_O_2_ (15%). At high concentrations exceeding 30% HNO_3_, only partial recovery of MPs is achieved. Recovery rates of 70% for nylon, 80% for PVC, and 90% for PE were established using 20% HNO_3_. The authors achieved full recovery of all polymers at HNO_3_ concentrations up to 10%; however, melted particles were nonetheless recorded. At 5% HNO_3_ and 15% H_2_O_2_, the tested polymers showed no visible changes, and the average recovery efficiency reached 98.0%. Yu et al. (2019) reported recovery rates of 90%–100% for seven different types of microplastics in mussel and fish samples using an HNO_3_:H_2_O_2_ mixture (4:1, 30 min, 50 °C). The reagents completely digested biotic samples with a mass below 5 g, with PET being the only polymer damaged.

While acidic methods act more aggressively on sensitive polymers, alkaline approaches are generally gentler for isolating MPs from biota (Süssmann et al., 2021). Fraissinet et al. (2021) established that a combination of 5% KOH and 5% H_2_O_2_ at 60 °C for 3 h, with the addition of 2.7% methanol, digests 99% of the soft tissues of *Mytilus galloprovincialis*. This protocol demonstrates high recovery of HDPE, PA, PP, PS, and PET without their degradation. Cole et al. (2014) compared 10 M NaOH with enzymatic and acidic methods and found that highly alkaline conditions induce the degradation or discoloration of PA, PVC, and PE. Similar results were reported by Hurley et al. (2018), who recorded mass reductions of PET and polycarbonate (PC) by 29% and 60%, respectively, following treatment with 10 M NaOH at 70 °C, as well as the depolymerization of PC. Simultaneously, a lower concentration (1 M NaOH) exhibits a significantly weaker impact on the polymers. Dehaut et al. (2016) demonstrated that NaOH (1 M and 10 M) can successfully eliminate biogenic matter such as zooplankton with up to 90% efficiency relative to weight loss, with no significant damage observed to the polymers, except for cellulose acetate (CA). However, using 40% NaOH (60 °C) resulted in the deformation of PA fibers, yellowing of PVC granules, and fusion of PE particles (Cole et al., 2014), while PC, CA, and PET were also damaged (Dehaut et al., 2016). As a compromise, the use of 10% KOH at 60 °C for 24 h is recommended, as it ensures the complete digestion of soft tissues (fish, mussels) while the impact on polymers remains limited (Dehaut et al., 2016; Dehaut et al., 2019).

Existing studies demonstrate high efficiency for enzymatic as well as acidic and alkaline approaches; however, to date, there is a lack of systematic comparison regarding their application to the same type of sample across different states. The selection of an appropriate method for the digestion of organic matter samples is conditioned by numerous factors, including the type, quantity, and state of the samples (fresh, frozen, or lyophilized), the types of polymers being analyzed, the required analysis time, and the research objectives. In this context, it is essential to achieve a balance between digestion efficiency and the preservation of the structural and chemical properties of the microplastics, which necessitates a careful selection of conditions and subsequent verification of their integrity.

The objective of the present study is to conduct a comparative assessment of the efficiency of enzymatic, acidic, and alkaline digestion protocols for the removal of organic matter from lyophilized and frozen *Mytilus galloprovincialis* specimens, within the context of sample preparation for microplastics analysis. Particular emphasis is placed on evaluating: (i) the digestion efficiency under controlled conditions, (ii) the influence of the physical state of the sample (lyophilized versus frozen) on digestion efficiency, (iii) the potential effects of the applied protocols on the chemical and morphological integrity of representative polymer types (HDPE, PA, PET, PVC), and (iv) the recovery of microplastics following the application of the optimal digestion method.

## 2 Materials and methods

### 2.1 Materials

Mediterranean mussels (*Mytilus galloprovincialis*) collected from the Gulf of Burgas, Black Sea, Bulgaria, were used as model bioindicators in the present study. To assess the impact of the various digestion protocols on microplastics, standard polymer particles with the following characteristics were used: PVC (100–200 μm), HDPE (100–500 μm), PA (100–200 μm), and PET (100–250 μm). The polymer particles used in this study were pristine commercial reference materials and were not environmentally weathered microplastics. The selected polymer particles were characterized prior to the digestion experiments to ensure consistent particle properties during the evaluation of digestion protocols. For enzymatic digestion, Kreon®25,000 (Abbott Laboratories GmbH, Germany) was used - a pharmaceutical product containing different pancreatic enzymes: 25,000 Ph. Eur units lipase, 18,000 Ph. Eur units amylase, and 1,000 Ph. Eur units protease. For the chemical protocols, the following reagents were used: potassium hydroxide KOH (90%, Merck), nitric acid HNO_3_ (65%, Merck), and hydrogen peroxide H_2_O_2_ (30%, Merck).

### 2.2 Sample preparation

**FIGURE 1:**
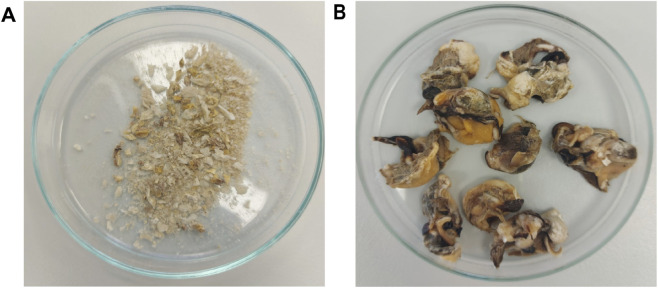
Lyophilized **(A)** and frozen **(B)** *Mytilus galloprovincialis* samples prior to digestion.

The *Mytilus galloprovincialis* samples were divided into two groups: lyophilized and frozen (Figure 1). Lyophilization was performed using a Labconco apparatus (model 70,020) at a temperature of −15 °C for 72 h under reduced vacuum (0.500 mbar) to eliminate free water. The frozen samples were stored at −20 °C until the commencement of the experiments. Soft tissues from the collected mussels were pooled and homogenized prior to the experiments in order to obtain a representative biological matrix for the digestion tests. The triplicate measurements performed for each experimental condition therefore represent technical replicates derived from the same homogenized tissue sample.

### 2.3 Biological matrix digestion

Samples of lyophilized (0.2 and 0.4 g) and frozen (2.0 and 4.0 g) mussels were subjected to three different digestion protocols: alkaline, acidic, and enzymatic. The selected sample masses were chosen to represent practical laboratory sample loads commonly used in microplastic monitoring studies while allowing a direct comparison between lyophilized and frozen tissue states.

#### 2.3.1 Alkaline digestion with KOH and H_2_O_2_

Each lyophilized and frozen mussel sample in the specified quantities was treated individually with a mixture of 20 mL of 10% KOH and 1 mL of 30% H_2_O_2_ at a temperature of 60 °C for 60 min, under constant stirring (400 rpm).

#### 2.3.2 Acidic digestion with HNO_3_ and H_2_O_2_

Acidic digestion was performed by adding 20 mL of HNO_3_ (65%) and 1 mL of H_2_O_2_ (30%) to the respective lyophilized or frozen mussel sample. The prepared mixture was incubated at 55 °C for 20 min with continuous stirring at 600 rpm.

#### 2.3.3 Enzyme digestion with Kreon®25,000

0.125 g of Kreon®25,000 (a pancreatic enzyme preparation) was dissolved in 20 mL of 1 M Tris-HCl buffer solution, with the pH adjusted to 8.0 - the optimal activity range for pancreatic enzymes (von Friesen et al., 2019; Berdutin et al., 2000). The samples were added to the enzyme solution and incubated at 37.8 °C for 2, 3, and 24 h under constant stirring (300 rpm). Lyophilized (0.2 and 0.4 g) and frozen (2.0 and 4.0 g) *Mytilus galloprovincialis* samples were processed separately.

### 2.4 Assessment of digestion efficiencies

To evaluate the effectiveness of the three protocols applied to the treatment of lyophilized and frozen mussels, the digestion efficiency parameter was determined. This parameter reflects the degree of dissolution of organic matter in the samples and serves as an indicator of the methodology’s efficiency in preparing samples for subsequent microplastics analysis. A gravimetric method was applied to calculate this parameter, based on the quantitative determination of the remaining undissolved organic matter post-treatment.

Prior to treatment, the mussel samples were weighed on a Kern analytical balance with a precision of 0.00001 g. Subsequently, the samples were subjected to alkaline, acidic, and enzymatic treatments to dissolve the organic matrix. Upon completion of the process, the resulting digestates were filtered through a pre-weighed metal filter (5 μm pore size). To monitor potential laboratory or airborne contamination, procedural blanks were included and processed following the same digestion and filtration procedures but without biological material. The blank filters were examined microscopically together with the experimental samples to verify the absence of contaminant particles introduced during laboratory handling. After filtration, the filters were rinsed with distilled water, placed in clean glass Petri dishes with lids, dried completely at 42 °C for 60 h, and reweighed. The digestion efficiency (%) was calculated by comparing the mass of the insoluble residue (the difference between the filter mass after and before treatment) to the initial sample mass using a modified formula by Maryani et al. (2020):

$$\text{Digestion efficiency }\left(\%\right)=\left(1-\frac{W_{\text{after}}-W_{\text{before}}}{W_{m}}\right)\times 100$$

where: *W*_m_ represents the initial mass of the sample (mussels) subjected to alkaline, acidic, or enzymatic digestion; *W*_before_ and *W*_after_ correspond respectively to the dry weights of the filter before filtration and the filter after digestion.

Additionally, the efficiency of each selected protocol was evaluated through visual microscopic inspection of the filters to identify any residual organic fragments that could potentially impede the detection of microplastics. The filters were analyzed under a BSCOPE BS.1153-EPLH microscope (Euromex, Holland) at ×40 magnification. The captured images were subjected to further visual analysis to confirm the completeness of the organic matrix digestion. All digestion experiments were performed in triplicate (n = 3 technical replicates per experimental condition). Each replicate consisted of an independently weighed subsample processed through the complete digestion and filtration procedure. The reported values represent the mean ± standard deviation (SD).

### 2.5 Assessment of the effects of digestion protocols on polymers

In order to evaluate the potential impact of the protocols applied for *Mytilus galloprovincialis* sample preparation on various types of plastics, four representative micropolymers were selected: PVC, HDPE, PA, and PET. Each polymer type was tested using the three implemented digestion methods: alkaline, acidic, and enzymatic. The treatment procedures followed the conditions described in the “Biological Matrix Digestion” section, but without the addition of biological material.

Prior to treatment, the polymer particles were photographed using a BSCOPE BS.1153-EPLH microscope to document color, surface texture, and general morphology. ATR-FTIR spectroscopy was used to confirm the chemical identity of the tested polymers and to evaluate potential structural changes after the applied digestion treatments. For each polymer group, ATR-FTIR spectra were recorded using an ALPHA II-P spectrometer (Bruker Optik GmbH, Germany) equipped with a Platinum ATR module and a diamond crystal (high-pressure version). Spectra were collected in the range of 4,000–400 cm^−1^ with a resolution of 4 cm^−1^ and averaged over 32 scans to improve the signal-to-noise ratio. The resulting spectra of the untreated micropolymers serve as references for subsequent comparison after treatment. Following treatment according to the respective protocols, the plastic particles were isolated, rinsed with filtered deionized water, dried, and re-analyzed both microscopically (for changes in color, transparency, and surface morphology) and spectroscopically. The post-treatment ATR-FTIR spectra were baseline-corrected and normalized to the intensity of the main absorption bands before comparison with the untreated control samples. This ensures reliable identification of any chemical changes in the polymer structure occurring as a result of the applied processing protocols.

For the quantitative assessment of the degree of structural changes post-treatment, the Hit Quality Index (HQI) was calculated - defined as the absolute value of the Pearson correlation coefficient (r) between the normalized spectra, expressed as a percentage. HQI values close to 100 indicate high spectral similarity and the absence of significant changes in the primary structure of the polymer (Weisser et al., 2022; Cowger et al., 2020).

### 2.6 Evaluation of microplastics recovery following the application of the optimal digestion protocol

Based on the results of the preliminary testing of the three digestion protocols, the selected optimal method was subjected to a validation procedure by determining the recovery rates of various types of microplastic particles. The objective of this analysis is to evaluate the efficiency of the protocol in degrading organic matter while simultaneously preserving and isolating microplastics for subsequent analysis.

To assess mass-based recovery, frozen *Mytilus galloprovincialis* tissue (2.0 g) was spiked with known masses of microplastic particles representing four polymer types: PVC, HDPE, PA, and PET (particle size ranges: 100–200 μm for PVC and PA, 100–500 μm for HDPE, and 100–250 μm for PET). Subsequently, the samples were subjected to treatment according to the conditions of the selected optimal enzymatic protocol. Upon completion of the digestion process, the resulting solutions were filtered through pre-weighed metal filters (5 μm pore size). In parallel, a control mussel sample without added polymers was processed using the same protocol and filtration. The control sample serves to account for the mass of the residual undigested organic matter remaining on the filter after digestion.

Since the degree of biological matrix degradation for the enzymatic protocol is 99.8%, it was noted that approximately 0.2% of the initial mussel mass remains as an undigested organic residue. This residue is accounted for indirectly through the use of the control sample.

The polymer recovery was calculated using a mass-corrected approach according to the formula:

$$\text{Recovery }\left(\%\right)=\left(\frac{W_{\text{spiked}}-W_{\text{control}}}{W_{\text{polymer}}}\right)\times 100$$

where: *W*_spiked_ is the mass of the filter after digestion of the sample with the added polymer; *W*_control_ is the mass of the filter after digestion of the control sample (without polymer); *W*_polymer_ is the initial mass of the added polymer.

This approach allows for the elimination of the influence of residual biomass and ensures a realistic assessment of microplastics recovery after treatment. The obtained recovery values were used to evaluate the effectiveness of the enzymatic protocol regarding the isolation of different types of micropolymers from the biological matrix.

### 2.7 Statistical analysis

To evaluate whether significant differences exist between the efficiency of the enzymatic, acidic, and alkaline treatments, Welch’s two-sample t-tests were performed. Digestion efficiency values were analyzed both separately for lyophilized and frozen samples, as well as in combination. Mean values and standard deviation (SD) were calculated, with the SD being used to assess the variability of results between individual replicates. To compare the methods, a two-way analysis of variance (ANOVA) was applied, with “Digestion method” and “Sample state” (lyophilized or frozen) as factors. All statistical calculations were performed using Python software (SciPy v.1.11.1), with a p-value <0.05 considered to indicate a statistically significant difference.

## 3 Results and discussion

To evaluate the efficiency and applicability of various biota digestion protocols for microplastics analysis, experiments were conducted using three of the most commonly employed methods: enzymatic, acidic, and alkaline treatments. The study included both lyophilized and frozen Mediterranean mussel (*Mytilus galloprovincialis*) samples, in quantities of 0.2, 0.4 g and 2.0, 4.0 g, respectively, allowing for a comparison of how different matrices behave. The following subsections present the results for each method individually, followed by a comparative analysis of their efficiency, validation through microscopic observation of the filters, and an assessment of the impact on the polymer structure of the microplastics. Finally, microplastic recovery is discussed as a key indicator of the practical applicability of the selected optimal protocol.

### 3.1 Efficiency of enzymatic digestion in lyophilized and frozen *Mytilus galloprovincialis* samples

For the enzymatic digestion of the mussel samples, a pharmaceutical enzyme preparation (Kreon®25,000) was utilized with three different incubation times - 2, 3, and 24 h. The resulting digestion efficiency values are presented in Table 1 and compared across the two sample types.

**TABLE 1:** Digestion efficiency of lyophilized and frozen *Mytilus galloprovincialis* samples following enzymatic, acidic, and alkaline treatments.

Digestion method	Temperature (°C)	Sample type	Time (h)	Sample mass (g)	Efficiency (%)
Enzymatic (Kreon®25000)	37.8	Lyophilized	2	0.2	98.20
Enzymatic (Kreon®25000)	37.8	Lyophilized	2	0.4	99.05
Enzymatic (Kreon®25000)	37.8	Lyophilized	3	0.2	98.50
Enzymatic (Kreon®25000)	37.8	Lyophilized	3	0.4	98.40
Enzymatic (Kreon®25000)	37.8	Lyophilized	24	0.2	98.99
Enzymatic (Kreon®25000)	37.8	Lyophilized	24	0.4	98.50
Enzymatic (Kreon®25000)	37.8	Frozen	2	2.0	99.80
Enzymatic (Kreon®25000)	37.8	Frozen	2	4.0	99.60
Enzymatic (Kreon®25000)	37.8	Frozen	3	2.0	98.60
Enzymatic (Kreon®25000)	37.8	Frozen	3	4.0	99.70
Enzymatic (Kreon®25000)	37.8	Frozen	24	2.0	99.88
Enzymatic (Kreon®25000)	37.8	Frozen	24	4.0	99.94
Acidic (HNO_3_ + H_2_O_2_)	55	Lyophilized	0.33	0.2	99.50
Acidic (HNO_3_ + H_2_O_2_)	55	Lyophilized	0.33	0.4	99.12
Acidic (HNO_3_ + H_2_O_2_)	55	Frozen	0.33	2.0	99.86
Acidic (HNO_3_ + H_2_O_2_)	55	Frozen	0.33	4.0	99.48
Alkaline (KOH + H_2_O_2_)	60	Lyophilized	1	0.2	96.74
Alkaline (KOH + H_2_O_2_)	60	Lyophilized	1	0.4	99.62
Alkaline (KOH + H_2_O_2_)	60	Frozen	1	2.0	99.94
Alkaline (KOH + H_2_O_2_)	60	Frozen	1	4.0	99.87

Enzymatic digestion of lyophilized *Mytilus galloprovincialis* showed high efficiency across all tested durations (2, 3, and 24 h). The mean degree of degradation ranged between 98.45% and 98.75%, regardless of the sample mass (0.2 and 0.4 g), suggesting that mass does not negatively impact enzymatic efficiency. The highest value was recorded at 24 h - 98.99%. This indicates that even the shorter incubation time (2 h) is sufficient for nearly complete digestion of the biota. Increasing the duration did not significantly improve the digestion efficiency further, which highlights the stability and reproducibility of the enzymatic method.

When comparing the effects of enzymatic treatment with Kreon®25,000 on lyophilized and frozen Mediterranean mussel samples (Table 1), it was found that both types underwent effective digestion—exceeding 98%. However, frozen samples demonstrated a higher degree of degradation, ranging between 98.60% and 99.94%, depending on the incubation time. At 2 h, degradation in frozen samples reached an average of 99.70%, while in lyophilized samples, it was 98.63%. As the duration increased to 24 h, the differences in the degree of degradation diminished, with maximum efficiency reaching 99.91% for frozen versus 98.75% for lyophilized samples.

The differences in enzymatic digestion efficiency between lyophilized and frozen *Mytilus galloprovincialis* can be explained by changes occurring in the tissue structure (Catarino et al., 2017) as a result of the storage method and/or pre-treatment (Brander et al., 2020). Lyophilization ensures almost complete removal of water while preserving the chemical composition of the sample (Hua et al., 2010), but it leads to the formation of a dry and sometimes more compact matrix, which may partially limit the penetration of enzymes or reagents. Furthermore, lyophilized samples typically require rehydration to achieve optimal enzymatic activity, explaining their lower degree of degradation compared to frozen samples.

In contrast, frozen mussel samples are more susceptible to degradation because tissue freezing significantly alters lipid and protein structures (Rosales et al., 2024). This likely facilitates biomass accessibility and the activity of the pancreatic enzymes in Kreon®25000 (primarily lipase, protease, and amylase). During incubation, the enzymes penetrate the tissue more rapidly, thereby degrading the biomass more effectively. The latter provides an advantage to frozen samples regarding the degree of degradation, as confirmed by Catarino et al. (2017). These observations are supported by the present study, where frozen samples showed higher digestion efficiency values across all tested protocols. This suggests that the physical state of the sample is a critical factor that must be considered in studies related to the extraction of microplastics from biota.

Compared to other protocols, which show efficiencies of: 97.7% ± 0.2% dry weight loss using Kreon®40,000 in Tris buffer at 37.5 °C overnight (von Friesen et al., 2019); >97% dry weight loss with Proteinase-K (50 °C, 2 h) (Cole et al., 2014); and 88% ± 2.5% wet weight loss using trypsin (40 °C, 30 min) (Courtene-Jones et al., 2017), the enzymatic protocol applied in this study demonstrates superior efficiency, exceeding 98% for both sample types within a 2-h period.

### 3.2 Efficiency of acidic digestion in lyophilized and frozen *Mytilus galloprovincialis* samples

The application of a combination of nitric acid and hydrogen peroxide resulted in high digestion efficiency for both types of analyzed samples (Table 1). For lyophilized mussels, the efficiency reached 99.50% for 0.2 g samples and 99.12% for 0.4 g samples. Frozen samples (2 g and 4 g) demonstrated degradation rates of 99.86% and 99.48%, respectively. The slightly higher values in frozen specimens (up to 99.86%) compared to lyophilized ones (up to 99.5%) can be attributed to the higher water content in the matrix, which facilitates the penetration and interaction of the acid with organic components. These results are in full agreement with those of Catarino et al. (2017), who associated higher digestion efficiency when treating with 35% (v/v) HNO_3_ for 1 h at 60 °C with the use of frozen samples and mechanical stirring.

The data from the present study confirm that the combination of HNO_3_ and H_2_O_2_ ensures high digestion efficiency (Bianchi et al., 2020; Yu et al., 2019) and is a reliable method for degrading the organic matrix (*Mytilus galloprovincialis*) within 30 min, with the sample mass having a minimal impact on the final efficiency. Despite the high effectiveness of the protocol in removing mussel tissue, strong acidic conditions may pose a risk for structural alterations in certain sensitive polymers. Lower nitric acid concentrations were not evaluated within the scope of the present study, as the aim was to assess the performance of a strong chemical digestion protocol commonly used in laboratory sample preparation. However, harmonized protocols for microplastic analysis generally recommend milder oxidative or enzymatic digestion procedures when preservation of polymer integrity is a priority (Bessa et al., 2019). Consistent with our ATR-FTIR and HQI results, which indicate measurable spectral changes in PA and PVC after acidic treatment, the use of concentrated HNO_3_ should therefore be applied with caution when acid-sensitive polymers are expected in the analyzed samples. Consequently, additional confirmation of polymer preservation after treatment is required (Section 3.6).

### 3.3 Efficiency of alkaline digestion in lyophilized and frozen *Mytilus galloprovincialis* samples

Alkaline digestion using a combination of potassium hydroxide (KOH) and hydrogen peroxide (H_2_O_2_) at 60 °C for 60 min showed high efficiency across nearly all samples, with the exception of one group of lyophilized samples. For the lyophilized samples, efficiency ranged between 96.74% (0.2 g sample) and 99.62% (0.4 g sample). The significantly lower value for the 0.2 g sample indicates protocol instability when dealing with small quantities of dry biomass. This may be due to uneven reagent penetration or limited diffusion within the more compact lyophilized matrix, suggesting that the method may be more sensitive to both sample mass and state.

The results of the alkaline treatment (Table 1) show nearly complete degradation of organic matter in the frozen samples - 99.94% for 2.0 g and 99.87% for 4.0 g. This indicates that alkaline digestion with an oxidizing agent is effective even for larger quantities of biomass from both lyophilized and frozen mussels. It is noted that the frozen samples again exhibited a slightly higher degree of degradation, likely due to better permeability and diffusion of the alkaline reagent into the tissue structure, consistent with observations from the enzymatic and acidic treatments.

While alkaline digestion is generally characterized by very high efficiency, the results show that variations can occur with small amounts of lyophilized biomass. This highlights the need for condition optimization and careful verification when working with smaller sample sizes. The method demonstrates potential for application in extracting microplastics from biota, maintaining high efficiency at larger masses and short reaction times.

### 3.4 Comparison of protocol efficiency for the digestion of lyophilized and frozen *Mytilus galloprovincialis* samples

**FIGURE 2:**
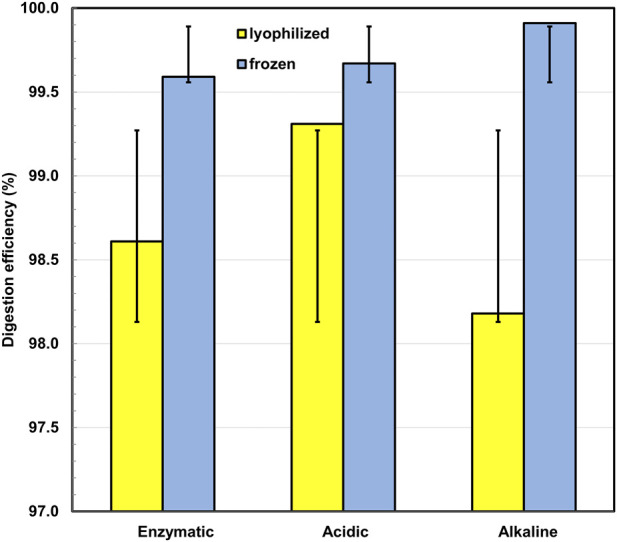
Mean efficiency (%) of enzymatic, acidic, and alkaline digestion methods applied to lyophilized and frozen *Mytilus galloprovincialis* samples. Yellow bars represent lyophilized samples, while blue bars indicate frozen samples. Error bars represent standard deviation (SD) of triplicate measurements.

The results of the comparative analysis indicate that all three implemented protocols demonstrate a high degree of efficiency (>96%) in degrading *Mytilus galloprovincialis*, in both lyophilized and frozen forms (Figure 2; Table 2). The differences observed between the tested digestion protocols are likely related to their different abilities to degrade biological matrices while preserving the integrity of the tested polymer particles. The highest stability in results was recorded during acidic treatment with a mixture of nitric acid and hydrogen peroxide, where the digestion rates showed very close values - 99.31% and 99.67% for lyophilized and frozen samples, respectively. The acidic method provides a comparable or even higher degree of degradation within a significantly shorter period (20 min) compared to enzymatic treatment, which requires 2–24 h to achieve results comparable to the chemical protocols. A decrease in efficiency was noted during the alkaline treatment of lyophilized samples, where the average digestion rate was 98.18%.

**TABLE 2:** Mean digestion efficiency and standard deviation (SD) of enzymatic, acidic, and alkaline treatments applied to *Mytilus galloprovincialis* samples. “All” represents the combined dataset including both lyophilized and frozen samples. The number of measurements corresponds to the number of experimental conditions included in the statistical comparison.

Digestion method	Sample type	Mean efficiency (%)	SD	Number of measurements
Enzymatic (Kreon®25000)	All	99.10	0.65	12
Enzymatic (Kreon®25000)	Lyophilized	98.61	0.34	6
Enzymatic (Kreon®25000)	Frozen	99.59	0.50	6
Acidic (HNO_3_ + H_2_O_2_)	All	99.49	0.30	4
Acidic (HNO_3_ + H_2_O_2_)	Lyophilized	99.31	0.27	2
Acidic (HNO_3_ + H_2_O_2_)	Frozen	99.67	0.27	2
Alkaline (KOH + H_2_O_2_)	All	99.04	1.54	4
Alkaline (KOH + H_2_O_2_)	Lyophilized	98.18	2.04	2
Alkaline (KOH + H_2_O_2_)	Frozen	99.91	0.05	2

Statistical analysis revealed that all three methods demonstrate similar efficiency - Table 2. The mean efficiency was 99.10% ± 0.65 (SD) for enzymatic, 99.49% ± 0.30 (SD) for acidic, and 99.04% ± 1.54 (SD) for alkaline treatment. The higher standard deviation observed in the alkaline digestion is primarily due to the lower efficiency in small-mass (0.2 g) lyophilized samples, indicating a higher sensitivity of this protocol to the sample state and biomass quantity. The differences between the protocols are not statistically significant (p > 0.05), confirming the comparability of the three approaches regarding final efficiency, despite variations in processing time and result stability. While all three digestion protocols demonstrated high efficiency in removing the biological matrix, their practical suitability differs when polymer preservation is considered. Chemical digestion, particularly under strong oxidizing acidic conditions, can provide rapid matrix removal but may increase the risk of structural alterations in more sensitive polymers such as PA and PVC. In contrast, enzymatic digestion requires longer incubation times but represents a more conservative sample preparation approach that minimizes potential damage to polymer particles and allows reliable downstream identification of microplastics.

Table 3 presents the results of the Welch’s two-sample t-tests and the two-way ANOVA. The values indicate that both in the pooled data and the stratified analysis by sample state, frozen mussels showed a trend toward slightly higher efficiency (up to 99.91%) compared to lyophilized ones. The two-way ANOVA confirmed that neither the digestion method nor the sample state had a statistically significant effect on the final efficiency (p > 0.05). Only in the comparison between enzymatic and acidic digestion of lyophilized samples was a trend toward higher efficiency of the acidic method observed (t = −2.99, p = 0.085); however, this did not reach statistical significance.

**TABLE 3:** Statistical comparison of digestion efficiency among enzymatic, acidic, and alkaline treatments applied to *Mytilus galloprovincialis* samples using t-tests and two-way ANOVA.

Comparison	t	p-value	Interpretation
Enzymatic vs. Acidic (All)	−1.63	0.130	Not significant (p > 0.05)
Enzymatic vs. Alkaline (All)	0.07	0.949	Not significant (p > 0.05)
Acidic vs. Alkaline (All)	0.57	0.606	Not significant (p > 0.05)
Enzymatic vs. Acidic (Lyophilized)	−2.99	0.085	Near significance (p ≈ 0.05)
Enzymatic vs. Alkaline (Lyophilized)	0.29	0.817	Not significant (p > 0.05)
Acidic vs. Alkaline (Lyophilized)	0.78	0.576	Not significant (p > 0.05)
Enzymatic vs. Acidic (Frozen)	−0.30	0.781	Not significant (p > 0.05)
Enzymatic vs. Alkaline (Frozen)	−1.54	0.181	Not significant (p > 0.05)
Acidic vs. Alkaline (Frozen)	−1.22	0.428	Not significant (p > 0.05)

### 3.5 Assessment of digestion efficiency through visual microscopic inspection of filters

**FIGURE 3:**
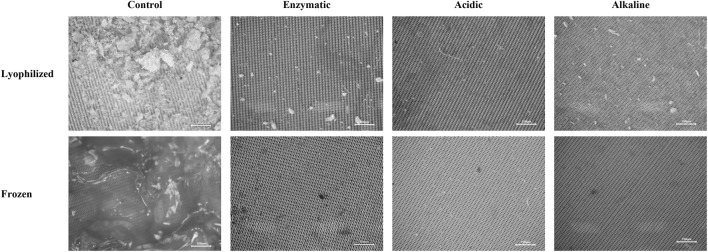
Representative microscopic images of filters from lyophilized and frozen *Mytilus galloprovincialis* samples after control, enzymatic, acidic, and alkaline digestion. Columns indicate the digestion treatment, while rows represent the sample preparation method. All images were acquired at ×40 magnification. Scale bar: 150 µm.

Additionally, the efficiency of each protocol was assessed through visual microscopic inspection of the filters to identify any potential residual organic fragments (Figure 3).

The control sample served as a reference for comparison with the treated samples. Across all samples subjected to enzymatic, acidic, and alkaline digestion, nearly complete removal of organic matter was observed. Only minimal residues were detected, predominantly in the form of isolated particles most likely of inorganic or mineral origin.

The results of the microscopic inspection confirm the high efficiency of all three digestion protocols and emphasize the importance of visual verification as a complementary qualitative approach for assessing the extent of organic matrix removal. Visual control is particularly important, as gravimetric indicators do not always reflect local variations or residual fragments that may interfere with subsequent analyses.

Furthermore, microscopic inspection represents a critical step in confirming the suitability of sample preparation for microplastic analysis, including verification of the preservation of particle morphology and the recognizability of potential synthetic particles following chemical or enzymatic treatment.

### 3.6 Assessment of the effects of digestion protocols on polymers

Despite the high digestion efficiency of *Mytilus galloprovincialis* achieved by all three methods, confirming the preservation of the morphology and chemical composition of polymer particles via microscopic or spectroscopic techniques is crucial to avoid false-negative results in micropolymer determination. This is particularly important when using chemical reagents for biota treatment, as they could compromise the integrity of certain sensitive polymers.

#### 3.6.1 ATR-FTIR and HQI analysis

ATR-FTIR spectra and Hit Quality Index (HQI) values were used to assess potential chemical alterations in the polymer structures following enzymatic, acidic, and alkaline treatments.

##### 3.6.1.1 HDPE spectral stability after digestion treatments

**FIGURE 4:**
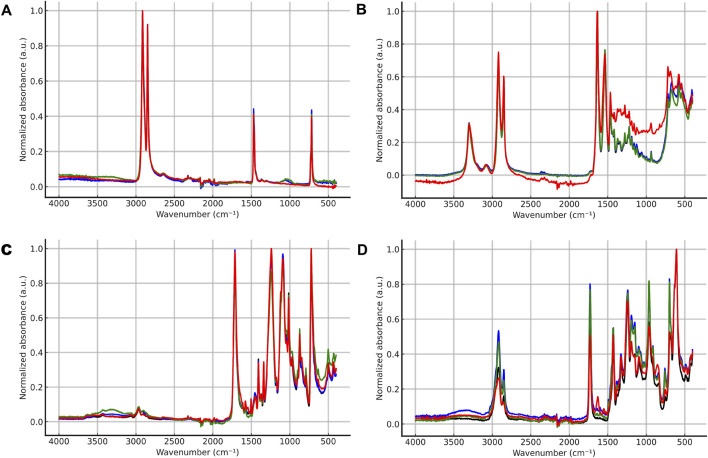
ATR-FTIR spectra of high-density polyethylene (HDPE) **(A)**, polyamide (PA) **(B)**, polyethylene terephthalate (PET) **(C)**, and polyvinyl chloride (PVC) **(D)** before and after the applied digestion treatment protocols. Black lines represent control samples, while blue, green, and red lines correspond to enzymatic, alkaline, and acidic digestion treatments, respectively. Spectra are shown as normalized absorbance versus wavenumber (cm^– 1^).

ATR-FTIR spectral analysis of HDPE particles before and after treatment (Figure 4A) reveals characteristic absorption bands for polyethylene: asymmetric and symmetric C–H stretching at 2,915 cm^−1^ and 2,847 cm^−1^, CH_2_ deformations at 1,472 cm^−1^, and rocking vibrations of long −(CH_2_)_n_− sequences at 720 cm^−1^. All observed peaks are consistent with reported literature data for HDPE (Circelli et al., 2024; Campanale et al., 2023). Figure 4A shows that regardless of the applied protocols (enzymatic, alkaline, or acidic), the spectra of the polyethylene particles maintain their spectral profiles without significant changes. The differences between them are minimal and show no signs of polymer degradation or the formation of new functional groups. The preservation of characteristic absorption bands at 2,915 and 2,847 cm^−1^, as well as the absence of new bands in the carbonyl (∼1710 cm^−1^) and hydroxyl (∼3,300 cm^−1^) regions, confirms that no oxidation or hydrolysis of the polyethylene chain occurs under the applied conditions.

Quantitative assessment of spectral similarity via HQI reveals an exceptionally high degree of similarity (over 99.2%) between all treated HDPE samples and the control - Table 4. These high HQI values confirm that the applied protocols do not induce significant chemical changes in the polyethylene structure. These findings demonstrate that enzymatic, acidic, and alkaline protocols do not affect the chemical integrity of polyethylene, which is consistent with its well-known chemical inertness resulting from the stable C–C and C–H bonds in the polymer chain (Schwab et al., 2024). Consequently, all three types of treatment can be considered compatible with polyethylene and can be applied without risk of chemical modification or degradation. Similar resistance of polyethylene to enzymatic and chemical treatments has been reported by other authors (Bianchi et al., 2020; Yu et al., 2019), who also found preservation of its characteristic FTIR bands and a lack of signs of chemical modification following acidic or alkaline treatments.

**TABLE 4:** Hit quality index (HQI) values for the analyzed polymers following enzymatic, acidic, and alkaline treatments compared to untreated controls.

Polymer type	Enzymatic HQI (%)	Acidic HQI (%)	Alkaline HQI (%)
HDPE	99.98	99.29	99.55
PA	99.98	93.68	99.77
PET	99.74	99.69	98.85
PVC	98.83	95.39	99.56

##### 3.6.1.2 PA spectral stability after digestion treatments

The ATR-FTIR spectra of PA microparticles (Figure 4B) show characteristic absorption bands typical of the amide groups in the nylon structure: amide I at approximately 1,650 cm^−1^ (C=O stretching), amide II at approximately 1,550 cm^−1^ (N–H deformations and C–N stretching), as well as a weak N–H stretching band in the 3,300–3,500 cm^−1^ region (Narayanan and Janardhanan, 2024; Smith, 2020). Quantitative assessment of spectral similarity (HQI) shows high values for enzymatic (99.98%) and alkaline treatments (99.77%), whereas for acidic treatment with HNO_3_, the HQI value drops significantly to 93.68% (Table 4). This decrease coincides with the observed reduction in intensity and slight shift of the amide I and amide II bands, suggesting partial hydrolysis of the amide bonds (Fan et al., 2025).

The results indicate that polyamide is more sensitive to chemical treatment, particularly in an acidic environment, whereas enzymatic and alkaline treatments do not lead to significant changes in its chemical structure. These findings clearly demonstrate that enzymatic and alkaline protocols do not compromise the chemical integrity of polyamide, while strong acids can induce partial degradation of amide bonds, making acidic treatment unsuitable for processing PA microplastics. Similar sensitivity of polyamides to acid hydrolysis has been reported in (Lusher et al., 2017).

##### 3.6.1.3 PET spectral stability after digestion treatments

The ATR-FTIR spectra of PET particles (Figure 4C) are consistent with published literature data for polyethylene terephthalate and display characteristic absorption bands typical of polyesters: a strong ester carbonyl (C=O) stretching band at around 1710 cm^−1^, aromatic C=C stretching near 1,600 cm^−1^, and C–O stretching in the 1,240–1,100 cm^−1^ region (Lujic et al., 2025). Following the treatments, PET retains its characteristic absorption bands without the appearance of new peaks or shifts that would indicate the destruction of the polymer backbone.

Quantitative assessment of spectral similarity via HQI shows high values for all treatments: enzymatic (99.74%), acidic (99.69%), and alkaline (98.85%) - Table 4. Although acidic treatment shows minimal spectral changes compared to the enzymatic one, a slight decrease in HQI is observed during alkaline treatment, likely due to the higher sensitivity of PET and partial alkaline hydrolysis of the ester groups (Donelli et al., 2010). These results show that PET exhibits high chemical resistance to enzymatic and acidic treatments, while an alkaline environment can induce limited hydrolysis of ester bonds. Therefore, enzymatic and acidic protocols can be considered compatible with PET, whereas prolonged alkaline exposure should be avoided when processing PET microplastics.

##### 3.6.1.4 PVC spectral stability after digestion treatments

ATR-FTIR spectra of PVC particles (Figure 4D) show characteristic absorption bands typical for this polymer: asymmetric and symmetric C–H stretching at 2,922 and 2,868 cm^−1^, CH_2_ deformations at 1,427 and 1,250 cm^−1^, and C–Cl stretching in the 600–700 cm^−1^ region (Fernández-Sanmartín et al., 2024). As observed, after enzymatic and alkaline treatment, the spectral profiles of the PVC samples remain nearly unchanged compared to the control sample, with no new peaks or significant shifts, confirming the preservation of the polymer’s chemical structure. Nevertheless, minor changes in the intensity of certain absorption bands were observed in both treatments, likely related to physical factors such as varying contact with the ATR crystal or surface effects, rather than actual chemical modifications. However, treatment with HNO_3_ resulted in a slight change in band intensity in the 1,500–1750 cm^−1^ region, as well as a weak shift in the C–Cl vibration region, which may be associated with partial dechlorination and/or surface oxidation of the PVC, leading to the formation of C=O and/or C=C bonds. This corresponds to the recorded decrease in HQI to 95.39% (Table 4), indicating that PVC is sensitive to the strong oxidative acidic conditions created by HNO_3_, whereas enzymatic and alkaline treatments do not cause significant structural changes. The results show that PVC exhibits high chemical stability under non-aggressive conditions, and enzymatic and alkaline protocols can be applied without risk of degradation. Similar sensitivity of PVC to acidic impact has been reported in (Fernández-González et al., 2022), noting that while surface oxidation is possible with strong acids, the primary polymer backbone remains largely unchanged.

In summary, the ATR-FTIR analysis and calculated HQI values demonstrate that the studied micropolymer types generally maintain their chemical structure after enzymatic and alkaline treatments, while measurable spectral changes were observed for PA and PVC after acidic digestion with HNO_3_. HDPE exhibited the highest chemical resistance, while PA proved to be the most sensitive to acidic treatment. PET and PVC showed minor changes related to partial hydrolysis of ester bonds (during KOH alkaline treatment) and surface oxidation (during HNO_3_ treatment), respectively. The findings confirm that enzymatic and alkaline protocols are compatible with all analyzed polymers, as they preserve their chemical integrity and allow for reliable subsequent identification of microplastics. In contrast, acidic treatment with HNO_3_ induces measurable spectral changes in PA and PVC, highlighting the need for caution when using this reagent. The summarized HQI values for all polymers are presented in Table 4.

#### 3.6.2 Visual and microscopic observations

Following the ATR-FTIR and HQI analyses, visual and microscopic observations were performed to complement the spectroscopic data and to assess potential morphological changes in the polymer particles after enzymatic, acidic, and alkaline digestion.

**FIGURE 5:**
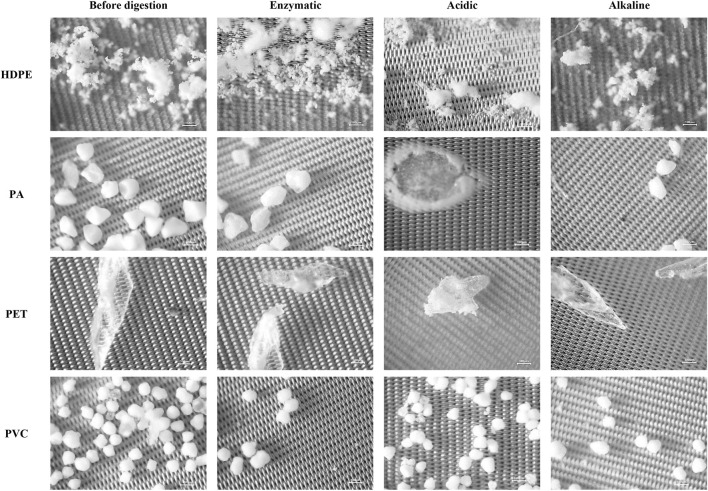
Representative microscopic images of micropolymer particles of HDPE, PA, PET, and PVC before digestion and after enzymatic, acidic, and alkaline treatments. Columns correspond to the digestion conditions, while rows represent the different polymer types. All images were acquired at ×40 magnification. Scale bar: 100 µm.

Figure 5 presents representative microscopic images of HDPE, PVC, PA, and PET micropolymer particles before and after the application of the three digestion protocols. Microscopic observations revealed that HDPE and PET particles maintain their shape, size, and surface integrity across all applied treatments. The particles remain clearly distinguishable on the filters, showing no signs of fragmentation, melting, or surface degradation. These results are consistent with the well-known high chemical resistance of polyolefins and polyesters to oxidative and alkaline environments, as reported by other authors applying chemical protocols for biota digestion (Fraissinet et al., 2021; Hurley et al., 2018).

In contrast, visible surface changes were observed in PA and PVC particles primarily after acidic treatment with HNO_3_. Comparable findings have been reported by Dehaut et al. (2016) and Hurley et al. (2018), who found that polyamides and PVC exhibit surface and spectral alterations following treatment with strongly oxidative acidic solutions. The sensitivity of PA to such environments is likely related to the presence of amide bonds, which are more susceptible to hydrolysis and oxidation compared to polyolefins.

Enzymatic and alkaline treatments did not induce visible morphological changes in any of the analyzed polymers - Figure 5. The particles retained their original shape and surface characteristics, indicating that these protocols do not lead to significant physical damage to the polymer materials. This is consistent with the findings of von von Friesen et al. (2019) and Catarino et al. (2017), which show that enzymatic and moderately alkaline protocols preserve the morphology and chemical structure of most microplastics.

The visual observations obtained are in full agreement with the ATR-FTIR results and high HQI values, confirming the preservation of the chemical identity of HDPE and PET under all treatments, as well as the absence of significant changes during enzymatic and alkaline treatment of PA and PVC. Previous studies have also reported that acidic treatment with HNO_3_ and other strong oxidants can induce surface changes in polymers such as PA and PVC (Cole et al., 2014; Claessens et al., 2013; Hurley et al., 2018), whereas alkaline treatment with KOH at moderate temperatures is compatible with most widely distributed microplastics (Dehaut et al., 2016).

The combined results from spectroscopic (ATR-FTIR and HQI) and microscopic analysis show that enzymatic and alkaline protocols preserve both the chemical and morphological integrity of the studied polymers, while acidic treatment can lead to partial surface alterations in more sensitive polymers such as PA and PVC. These changes are consistent with the observed spectral deviations and confirm the higher sensitivity of polymers containing polar or heteroatomic bonds to acidic environments. Similar recommendations are formulated in the harmonized JPI Oceans protocol (Bessa et al., 2019), which emphasizes the importance of selecting a digestion method based on the expected polymer composition of the samples.

Based on the observations made, the enzymatic protocol stands out as the most gentle regarding the preservation of the morphology and surface characteristics of the studied polymers, identifying it as the most suitable for the subsequent assessment of microplastic recovery in *Mytilus galloprovincialis* samples. The application of this method ensures a high degree of certainty that microplastic particles can be isolated without significant structural alterations. Therefore, the alkaline and acidic protocols were not used during the recovery determination stage, in order to avoid potential additional impacts of the reagents on the structure of the studied micropolymers. It should be emphasized that the applied treatments represent laboratory sample preparation procedures used for the removal of biological matrices prior to microplastic analysis and are not intended to simulate environmental degradation processes occurring in marine ecosystems.

### 3.7 Assessment of microplastic recovery following the application of the optimal digestion protocol

A key criterion for the reliability of any biological matrix digestion method is its ability to allow for the effective isolation and recovery of microplastic particles after processing. Based on the results from Sections 3.1–3.6, the enzymatic protocol using Kreon®25,000 was identified as optimal due to the high degree of organic matter degradation and the absence of observed morphological and chemical alterations in the studied polymers, as confirmed by ATR-FTIR, HQI, and microscopic analysis. Consequently, this specific protocol was used to assess microplastic recovery.

**FIGURE 6:**
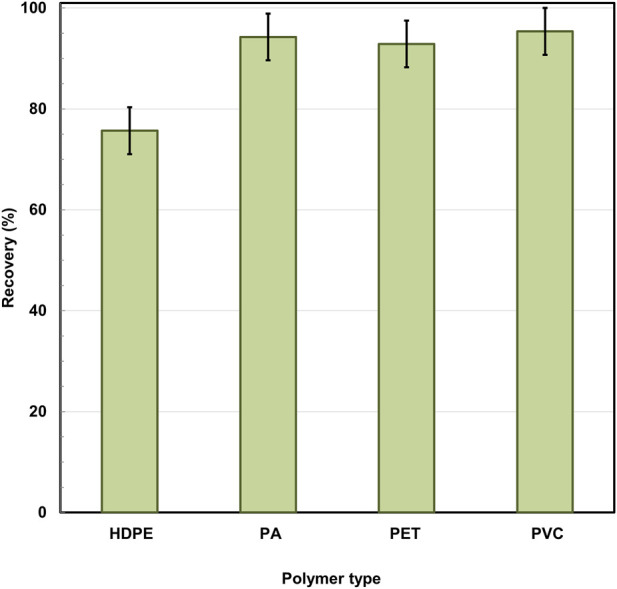
Polymer mass-corrected recovery (%) of selected polymers after enzymatic digestion of *Mytilus galloprovincialis* samples.

For this purpose, known quantities of four representative polymers - HDPE, PA, PVC, and PET were spiked into frozen *Mytilus galloprovincialis* samples, which were then subjected to enzymatic digestion under the described conditions. After filtration, recovery was assessed using a mass-corrected gravimetric approach, accounting for the residual undigested organic matter retained on the filter post-digestion. The results are presented in Figure 6, and the respective masses of the spiked and recovered polymers are summarized in Table 5.

**TABLE 5:** Added and recovered polymer masses used for the calculation of mass-corrected recovery after enzymatic digestion of *Mytilus galloprovincialis*.

Polymer	Added polymer mass (g)	Recovered polymer mass (g)	Recovery (%)
HDPE	0.01238	0.00937	75.69
PA	0.01547	0.01458	94.25
PET	0.00855	0.00794	92.87
PVC	0.01747	0.01665	95.36

The results show distinct differences in recovery rates depending on the polymer type. HDPE showed a recovery of 75.69%, while for PA, PET, and PVC, the values were significantly higher - 94.25%, 92.87%, and 95.36%, respectively. These results indicate that the enzymatic protocol enables the effective isolation of most of the studied polymers after the digestion of the biological matrix. The lower recovery rate for HDPE is likely due to the specific physicochemical characteristics of polyethylene, including its low density, hydrophobicity, and tendency to adhere to laboratory surfaces, which can lead to losses during transfer, rinsing, and filtration procedures (Dimante-Deimantovica et al., 2022). Such effects may result in partial particle losses during sample preparation. In addition, the HDPE particles used in this study covered the widest size range (100–500 μm), and potential size-dependent losses during processing cannot be excluded. Importantly, these differences in recovery are not associated with chemical or morphological degradation of the polymer, as ATR-FTIR and microscopic analyses confirmed the preservation of HDPE structure following enzymatic digestion.

The digestion efficiencies observed in this study correspond to values reported in earlier studies using enzymatic approaches for the isolation of microplastics from biological matrices. López-Rosales et al. (2022) report recovery rates in the range of 85%–92% for PET, PA, and PVC, and approximately 87% for PE during the enzymatic treatment of biological samples, with an overall particle recovery of around 88%. Karlsson et al. (2017) also report recovery rates of up to 97% when using an enzymatic approach (Proteinase-K) followed by spectroscopic identification of the polymers. In this context, the high recovery rates obtained for PA, PET, and PVC in the present study are consistent with previously reported findings, whereas the lower value observed for HDPE underscores the limitations of mass-based approaches when working with lightweight and hydrophobic polymers.

A comparison of Figure 6 and Table 5 shows that the mass-corrected approach allows for a realistic assessment of recovery and emphasizes the need to account for polymer-specific characteristics in the quantitative analysis of microplastics. The results confirm that enzymatic digestion represents a reliable and gentle method for analyzing microplastics in biological matrices, while simultaneously demonstrating the importance of correctly accounting for recovery across different polymer types.

A recovery control without biological matrix was not included in the present study. Such controls could help to quantify potential particle losses related to sample handling, transfer, or filtration procedures independently of biological matrix digestion and should be considered in future studies.

A limitation of the present study is that the experiments were conducted using pristine commercial polymer particles rather than environmentally weathered microplastics. In natural environments, microplastic particles may exhibit surface oxidation, biofouling, fragmentation, and aging-related chemical modifications that can influence their interaction with digestion reagents. Therefore, while the present results provide a controlled methodological comparison of digestion protocols, caution should be exercised when extrapolating the findings directly to environmentally aged microplastics.

## 4 Conclusion

The comparative evaluation of enzymatic, acidic, and alkaline digestion protocols demonstrated that all three approaches are highly effective for the removal of organic matter from *Mytilus galloprovincialis*, achieving digestion efficiencies above 96% for both lyophilized and frozen samples. Acidic digestion proved to be the fastest method, providing near-complete digestion within a short time, while alkaline digestion showed high efficiency but slight variability when applied to small amounts of lyophilized biomass. Enzymatic digestion, although slower, showed the highest stability and reproducibility of results. The physical state of the samples significantly influenced digestion performance. Frozen samples consistently exhibited slightly higher digestion efficiencies than lyophilized ones, suggesting that preserved tissue hydration improves reagent accessibility. Combined ATR-FTIR, HQI, and microscopic analyses confirmed that enzymatic and alkaline treatments preserve both the chemical structure and morphology of the tested polymers (HDPE, PA, PET, PVC). In contrast, acidic treatment induced visible and spectral surface alterations in PA and PVC, indicating potential risks for quantitative microplastic analysis when this protocol is applied. Recovery experiments further confirmed the suitability of the enzymatic protocol, with high mass-corrected recovery values for PA, PET, and PVC, and acceptable recovery for HDPE considering its physical properties. Overall, the enzymatic protocol with Kreon®25,000 is recommended as the most reliable and polymer-safe method for sample preparation in microplastic studies involving *Mytilus galloprovincialis*, as it ensures efficient organic matter removal while preserving the integrity and recoverability of microplastic particles.

## Data Availability

The original contributions presented in the study are included in the article/supplementary material, further inquiries can be directed to the corresponding author.
